# Editorial: The Role of Media in Suicide and Self-Harm: Cross-Disciplinary Perspectives

**DOI:** 10.3389/fpsyg.2022.932117

**Published:** 2022-05-24

**Authors:** Qijin Cheng, Yukari Seko, Thomas Niederkrotenthaler

**Affiliations:** ^1^Department of Social Work, The Chinese University of Hong Kong, Sha Tin, Hong Kong SAR, China; ^2^School of Professional Communication, Ryerson University, Toronto, ON, Canada; ^3^Unit Suicide Research & Mental Health Promotion, Department of Social and Preventive Medicine, Center for Public Health, Medical University of Vienna, Vienna, Austria

**Keywords:** suicide, self-harm, mass media, social media, prevention

Suicide and self-harm are complex, multifaceted, and simultaneously personal and social phenomena. While what motivates a person to engage in these acts cannot be reduced to a single factor, the media's role as a shaper and conduit of meanings has attracted considerable scholarly and practitioner attention. Although the mass media has been, and will doubtlessly continue to play a key role in shaping public attitudes and behaviors toward suicide and self-harm, the user-generated media has dramatically diversified our opportunities to encounter and interact with media content featuring these behaviors.

The nine articles in this Research Topic together provide a contemporary snapshot of this rapidly changing field. The articles cut across disciplinary boundaries, representing a wide range of perspectives from public health, psychiatry, psychology, cultural studies, communication studies, and computer science. The cross-disciplinary efforts demonstrate how researchers and practitioners across the world tackle the intricate role of the media in suicide and self-harm through their unique epistemological lens.

The media has long been viewed as a double-edged sword that can ameliorate or exacerbate suicide and self-harm. Mounting evidence suggests a considerable correlation between incautious media coverage—particularly the reporting of celebrity suicides—and perceived increase in suicidal behaviors (Niederkrotenthaler et al., 2020). Although guidelines for reporting suicide help media professionals improve in some areas of suicide coverage [e.g., World Health Organization (WHO), 2017], the old habit still clings. In this Research Topic, Ng et al. illuminate a shared concern among Malaysian stakeholders about unsafe suicide reporting. Media practitioners, mental health professionals, and people with lived experience of suicidal behaviors commonly concerned with sensational and emotionally provoking media coverage that presents suicide content as “newsworthy.”

In response to the persistent need for sensible suicide reporting, three articles in this collection explored educational interventions for key stakeholders. The safe reporting training for Malaysian media professionals examined by Lim et al. showed promising effects on trainees with no lived experience of suicidal behavior. Similarly, Walter et al. found their Mini Media training for French psychiatrists effective in improving psychiatrists' ability to guide journalists toward a more responsible reporting of suicide. Braun and Niederkrotenthaler conducted a randomized controlled trial to investigate if educative media material would influence Austrian physicians' attitudes to initiate life-saving procedures after a suicide attempt. Results showed little impact of the media-based education, as physicians tended to make a procedural decision based on their own opinions on suicide and specific patient case. Altogether, these articles indicate an expanding horizon of educational intervention from targeting solely on media professionals to including healthcare providers who advise journalists or support suicidal people. Future studies of this kind should take into account intervention targets' prior knowledge and attitude toward suicide.

Meanwhile, the expansion of social media requires suicide and self-harm research to extend its reach to this emergent field. Bell and Westoby argue that in our current “polymediated age” where anyone can participate in media communication as producers, consumers, audiences, and critics, exposure to suicide-related content has become much more complicated. Within the emergent “communicative ecology of exposure” (Bell and Westoby), social media are taking on vital roles and reproducing both benefits and harms of traditional media. Seong et al.'s study on Korean young adolescents with a history of self-harm highlighted the potential risk of social media use. They found that youth who have posted content about one's self-harm showed an increased risk of lifetime suicidality and suicide attempt.

In this regard, there is a growing excitement around Artificial Intelligence (AI) and machine learning technologies as a potential game changer in suicide prevention. Yang et al. reported on their AI-based “Tree Hole Action” program that monitors Chinese social media posts, detects individuals at risk for suicide, and then deploys trained volunteers to proactively offer interventions. However, while the study found promising results, some users may not feel comfortable being monitored or to have their family and friends alerted about what they posted on social media. The possibility of monitoring, predicting, and intervening in suicidal behaviors of social media users poses serious ethical questions (Ophir et al., 2021). Is the protection of life more important than any potential invasion of privacy? Future suicide and self-harm research should discuss further ethical dilemmas pertinent to digital health surveillance.

Finally, two contributions from communication and cultural scholars spur a focal shift from individual behaviors to group, community, and (sub)culture wherein meanings of suicide and self-harm are co-constructed and performed. Seko and Kikuchi's conceptual paper describes a cross-platform promulgation of “*menhera*” girl characters across Japanese popular culture (i.e., *manga*, game, fashion) that portray self-harm as a self-sufficient signifier of female “madness.” The authors argue that this gendered caricature, which simultaneously reproduces and disrupts traditional gender norms and stigma associated with self-harm, may provide people who self-harm with a convenient frame of reference to understand and describe their experiences beyond pathological interpretation. In studying an online pro-recovery suicide forum, Alvarez similarly contends that forum members ascribe alternative meanings to their suicidal self. For them, staying alive—or continuing to *be* in Heideggerian sense—depends not on reconstitution of a holistic (thus “normal”) self, but on active and ongoing reconciliation of fractured identities. The author suggests that clinical practice can benefit from recognizing the agency of suicidal people and involving them as active participants in their own recovery.

As we conclude this editorial, new and unprecedented issues continue to rise around the media's relationship to suicide and self-harm. Our intent has been to inspire cross-disciplinary dialogues that deepen our understanding of these two interrelated phenomena, challenge assumptions, and inspire new questions and interventions. It is our hope that researchers and practitioners working in suicide and self-harm would benefit from insights from different disciplines than their own and promote novel approaches to this expanding field of research and practice.

## Author Contributions

All authors listed have made a substantial, direct, and intellectual contribution to the work and approved it for publication.

## Conflict of Interest

The authors declare that the research was conducted in the absence of any commercial or financial relationships that could be construed as a potential conflict of interest.

## Publisher's Note

All claims expressed in this article are solely those of the authors and do not necessarily represent those of their affiliated organizations, or those of the publisher, the editors and the reviewers. Any product that may be evaluated in this article, or claim that may be made by its manufacturer, is not guaranteed or endorsed by the publisher.
